# Most Systematic Reviews and Meta-analyses Reporting Clinical Outcomes of the Remplissage Procedure Have at Least 1 Form of Spin

**DOI:** 10.1016/j.asmr.2024.100969

**Published:** 2024-06-29

**Authors:** Tom R. Doyle, Martin S. Davey, Thomas K. Moore, Max White, Eoghan T. Hurley, Christopher S. Klifto, Jonathan F. Dickens, Hannan Mullett

**Affiliations:** a The Royal College of Surgeons in Ireland, Dublin, Ireland; b UPMC Sports Surgery Clinic, Santry, Ireland; c Department of Orthopaedic Surgery, Duke University, Durham, North Carolina, U.S.A.

## Abstract

**Purpose:**

To determine the prevalence of spin in systematic reviews (SRs) and meta-analyses of clinical studies of the remplissage procedure.

**Methods:**

Two reviewers independently performed a literature search of the PubMed, Scopus, and Embase databases using the search term “remplissage” in accordance with Preferred Reporting Items for Systematic Reviews and Meta-analyses (PRISMA) guidelines. The full article of each included SR was assessed for the presence of the 15 most common types of spin. Methodologic quality was assessed using the second version of A Measurement Tool to Assess Systematic Reviews (AMSTAR 2).

**Results:**

A total of 15 SRs (8 accompanied by meta-analyses; 6 Level III and 9 Level IV) were included. Overall, 13 SRs (86.7%) contained at least 1 form of spin, with 33 unique instances of spin recorded; the mean frequency was 2.2 ± 1.3 (range, 0-4). The most prevalent form of spin, present in 11 studies (73%), was type 9 (“conclusion claims the beneficial effect of the experimental treatment despite reporting bias”). There were 14 uses of spin classified as misleading reporting, 16 classified as misleading interpretation, and 3 classified as inappropriate extrapolation. The mean 5-year impact factor of the publishing journals was 4.4 ± 0.9 (range, 0-6.1), the mean number of citations per SR was 33.3 ± 24.9 (range, 0-55), and the mean number of citations per month since publication was 0.68 ± 0.44 (range, 0-1.48). According to the AMSTAR 2 assessment, confidence in the results of the SRs was rated as critically low for 20% of reviews, low for 33.3%, and moderate for 46.7%.

**Conclusions:**

Most SRs of the remplissage procedure are affected by the presence of spin. Favorable reporting was observed in the absence of definite findings, as was minimization of drawbacks for certain populations.

**Level of Evidence:**

Level IV, systematic review of Level III and IV studies.

Arthroscopic Bankart repair (ABR) represents the most commonly performed procedure for anterior shoulder instability in the United States.^1^ Higher failure rates have been observed after ABR in shoulders with both on- and off-track Hill-Sachs lesions.^2^^,^^3^ Purchase et al.^4^ described the remplissage procedure, which involves capsulo-tenodesis of the infraspinatus tendon and posterior aspect of the capsule to fill the Hill-Sachs lesion to prevent engagement with the glenoid.^5^^,^^6^ A growing body of evidence suggests that remplissage significantly lowers recurrence rates compared with ABR alone.^3^^,^^7^ Remplissage is associated with a loss of external rotation, and although there is conflicting evidence regarding the clinical relevance of this, it is considered troublesome in overhead athletes.^8^, ^9^, ^10^ Remplissage has emerged as a potential alternative to bone block procedures in the absence of substantial glenoid bone loss, with fewer reported complications and similar recurrence rates and patient-reported outcomes.^9^^,^^11^ However, concerns persist that remplissage may lead to greater recurrence rates than bone block procedures in higher-risk groups, namely young collision athletes and patients undergoing revision surgery.^7^^,^^9^^,^^12^

Spin is a form of bias whereby positive findings are overstated and there is minimization of negative findings, which can distort the interpretation of studies.^13^, ^14^, ^15^ Studies of topics with a limited evidence base such as remplissage are often at risk of spin in their reporting, and therefore, systematic reviews (SRs), which collate data from these studies, may also be at risk. SRs play an important role in evidence-based medicine and are often used to inform surgical decision making.^16^, ^17^, ^18^ Because surgeons may often preferentially read abstracts over full texts, spin is particularly observed in abstracts owing to the incentive to strongly convey the authors’ message in a limited word count.^19^^,^^20^ Abstracts have been shown to display discrepancies with the findings of the full-text articles and high rates of reporting with spin, and the presence of spin has been shown to affect the interpretation of trial findings.^21^, ^22^, ^23^, ^24^

The purpose of this study was to determine the prevalence of spin in SRs and meta-analyses of clinical studies of the remplissage procedure. The hypothesis was that SRs reporting clinical results of the remplissage procedure would possess high rates of spin.

## Methods

### Study Selection

Two independent reviewers (M.W. and T.K.M.) performed a literature search of the PubMed, Embase, and Cochrane Library databases in January 2024 using the term “remplissage” in accordance with Preferred Reporting Items for Systematic Reviews and Meta-analyses (PRISMA) guidelines.^25^

### Eligibility Criteria

The inclusion criteria consisted of (1) SRs with or without accompanying meta-analyses with a primary aim to assess the use of remplissage for anterior shoulder instability, (2) publication in English in a peer-reviewed journal, and (3) availability of full-text article. The exclusion criteria consisted of all studies with methodologies other than SRs.

Duplicate titles were manually removed. For all remaining texts, the title and abstract were reviewed, and potentially eligible studies received a full-text review. The reference lists of the included studies found in the initial search were manually screened for additional articles meeting the inclusion criteria. The study protocol was not registered because it did not meet the International Prospective Register of Systematic Reviews (PROSPERO) inclusion criteria.

### Data Extraction and Analysis

The 2 independent reviewers extracted all relevant information using a predetermined data extraction sheet. The senior author (H.M.) was nominated to resolve any disagreements. Data that were extracted included study characteristics; country of origin; journal of publication, as well as its impact factor and recommendations regarding PRISMA and PROSPERO compliance; level of evidence; and methodologic quality of evidence (MQOE).

### Methodologic Quality of Evidence

The level of evidence of the included studies was evaluated based on the criteria described by *The Journal of Bone and Joint Surgery*.^26^ The MQOE of the included SRs was assessed using the validated second version of A Measurement Tool to Assess Systematic Reviews (AMSTAR 2).^27^ This is a 16-question tool that assesses the methodology of reviews and gives an overall rating of confidence in the findings of a review, based on the presence of weaknesses in critical domains, as follows: high, moderate, low, or critically low. Prior to data collection, both reviewers received training on the AMSTAR 2 from example data sets.^27^^,^^28^ Additionally, they underwent training to identify the 15 seminal types of spin as defined by Yavchitz et al.^13^ Spin is a form of reporting bias with minimization of negative findings and overemphasis of positive findings; there are 3 broad categories of spin: misleading reporting, inappropriate extrapolation, and misleading interpretation.^13^

### Statistics

Data were collected in Microsoft Excel and statistical tests were performed using open-source Jamovi software (version 2.3.28). Descriptive statistics were used to characterize the frequency of spin and AMSTAR 2 findings. Categorical and continuous variables were assessed using the χ^2^ test, Fisher test, or Student *t* test as appropriate. Multivariable linear regression analysis was used to assess the relation between study and journal characteristics with spin. *P* < .005 was considered statistically significant.

## Results

### Literature Search

**Fig 1:**
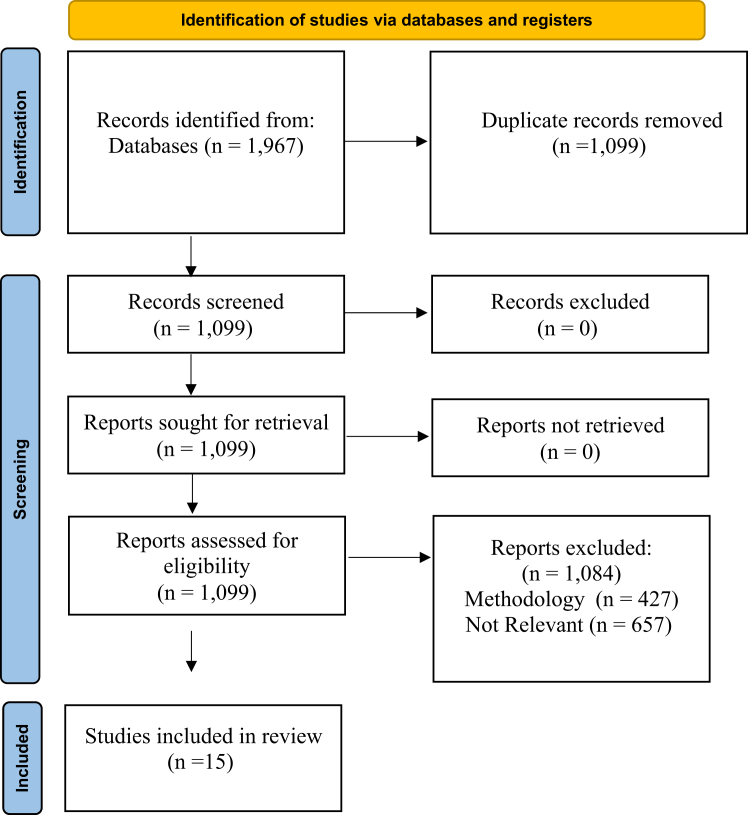
Preferred Reporting Items for Systematic Reviews and Meta-analyses (PRISMA) flowchart.

The search yielded 1,967 studies, with 1,099 remaining after removal of duplicates. After application of the inclusion and exclusion criteria, 15 studies were eligible for inclusion in our SR, as shown in Figure 1.

### Spin

Overall, 13 SRs (86.7%) contained at least 1 form of spin, with a mean spin frequency of 2.2 ± 1.3 (range, 0-4). There were 33 unique instances of spin recorded, of which 14 were classified as misleading reporting; 16, misleading interpretation; and 3, inappropriate extrapolation, as shown in Table 1. The most prevalent form of spin, present in 11 studies (73%), was type 9: “conclusion claims the beneficial effect of the experimental treatment despite reporting bias.” There was no significant difference in the prevalence of spin between studies reporting potential conflicts and studies reporting no potential conflicts (2.8 ± 1.1 and 1.9 ± 1.3, respectively; *P* = .021) or based on AMSTAR 2 rating (*P* = .113). On linear regression analysis, there was no association between journal impact factor and the prevalence of spin (*P* = .392).

**Table 1:** Frequency of Type and Category of Spin

Category	Type	Description and Citation^13^	No. of Instances of Spin	% of Studies
Misleading reporting			14	—
	3	Selective reporting of or overemphasis on efficacy outcomes or analysis favoring beneficial effect of experimental intervention	1	7
	5	Conclusion claims beneficial effect of experimental treatment despite high risk of bias in primary studies	10	67
	6	Selective reporting of or overemphasis on harm outcomes or analysis favoring safety of experimental intervention	1	7
	10	Authors hide or do not present any conflict of interest	0	0
	11	Conclusion focuses selectively on statistically significant efficacy outcome	1	7
	13	Failure to specify direction of effect when it favors control intervention	0	0
	14	Failure to report wide confidence interval of estimate	1	7
Misleading interpretation			16	—
	1	Conclusion contains recommendations for clinical practice not supported by findings	1	7
	2	Title claims or suggests beneficial effect of experimental intervention not supported by findings	0	0
	4	Conclusion claims safety based on non–statistically significant results with wide confidence interval	1	7
	9	Conclusion claims beneficial effect of experimental treatment despite reporting bias	11	73
	12	Conclusion claims equivalence or comparable effectiveness for non–statistically significant results with wide confidence interval	3	20
Inappropriate extrapolation			3	—
	7	Conclusion extrapolates review’s findings to different intervention	0	0
	8	Conclusion extrapolates review’s findings from surrogate marker or specific outcome to global improvement of disease	0	0
	15	Conclusion extrapolates review’s findings to different population or setting	3	0

### Study and Journal Characteristics

Overall, 15 SRs were included in the analysis, of which 8 were accompanied by meta-analyses. The SRs were published in 7 different journals between 2013 and 2024, as shown in Table 2. There were 5 publications in *Arthroscopy*^5^^,^^6^^,^^29^, ^30^, ^31^, ^32^, ^33^; 2 in the *Journal of Shoulder and Elbow Surgery*^34^^,^^35^; 2 in *Knee Surgery, Sports Traumatology, Arthroscopy*^36^^,^^37^; and 1 each in *The American Journal of Sports Medicine*,^38^ *The Journal of Bone and Joint Surgery*,^39^ *JSES Reviews, Reports, and Techniques*,^40^ and *Orthopaedics & Traumatology: Surgery & Research*.^41^ The mean 5-year impact factor of the publishing journals was 4.4 ± 0.9 (range, 0-6.1). The mean number of citations per SR was 33.3 ± 24.9 (range, 0-55), and the mean number of citations per month since publication was 0.68 ± 0.44 (range, 0-1.48), as shown in Table 3. There was no significant relation between the number of instances of spin and the 5-year impact factor (*P* = .99) or the number of citations per month (*P* = .191).

**Table 2:** Journal Publishing Information

Journal	No. of Publications	5-yr Impact Factor	PRISMA Required	PROSPERO Recommended
Arthroscopy	7	4.6	Yes	Yes
JSES	2	3.3	Yes	No
KSSTA	2	3.9	No	No
AJSM	1	6.1	No	No
JBJS	1	5.9	Yes	No
JSES RRT	1	None	Yes	No
OTSR	1	2.7	Yes	Yes

AJSM, *The American Journal of Sports Medicine*; JBJS, *The Journal of Bone and Joint Surgery* (American); JSES, *Journal of Shoulder and Elbow Surgery*; JSES RRT, *JSES Reviews, Reports, and Techniques*; KSSTA, *Knee Surgery, Sports Traumatology, Arthroscopy*; OTSR, *Orthopaedics & Traumatology: Surgery & Research*; PRISMA, Preferred Reporting Items for Systematic Reviews and Meta-analyses; PROSPERO, International Prospective Register of Systematic Reviews.

**Table 3:** Characteristics of Included Systematic Reviews

Authors	Year	LOE	Journal	Citations	Citations per Month	PRISMA Compliant	PROSPERO Compliant	Funding Source	AMSTAR 2 Rating
Villarreal-Espinosa et al.^36^	2024	III	KSSTA	0	NA	Yes		None	Low
Schrouff and Verlaan^40^	2023	III	JSES RRT	0	0	Yes		None	Moderate
Davis et al.^38^	2023	IV	AJSM	1	0.33	Yes		None	Low
Gouveia et al.^29^	2023	IV	*Arthroscopy*	3	0.33	Yes		None	Low
Gouveia et al.^30^	2021	IV	*Arthroscopy*	23	0.66	Yes		None	Moderate
Haroun et al.^34^	2020	III	JSES	40	1.48	Yes	Yes	None	Moderate
Hurley et al.^35^	2020	III	JSES	41	1.11	Yes		None	Moderate
Alkaduhimi et al.^31^	2019	III	*Arthroscopy*	28	0.49	Yes		None	Moderate
Lazarides et al.^6^	2019	IV	*Arthroscopy*	76	1.29	Yes		None	Moderate
Camus et al.^41^	2018	III	OTSR	38	0.6	Yes		None	Critically low
Liu et al.^5^	2018	IV	*Arthroscopy*	67	1.06	Yes		None	Moderate
Rashid et al.^37^	2016	IV	KSSTA	22	0.23			None	Low
Longo et al.^33^	2014	IV	*Arthroscopy*	49	0.45	Yes		None	Critically low
Buza et al.^39^	2014	IV	JBJS	55	0.47			None	Critically low
Leroux et al.^32^	2013	IV	*Arthroscopy*	55	1.01			None	Low

AJSM, *The American Journal of Sports Medicine*; AMSTAR 2, second version of A Measurement Tool to Assess Systematic Reviews; JBJS, *The Journal of Bone and Joint Surgery* (American); JSES, *Journal of Shoulder and Elbow Surgery*; JSES RRT, *JSES Reviews, Reports, and Techniques*; KSSTA, *Knee Surgery, Sports Traumatology, Arthroscopy*; LOE, level of evidence; NA, not applicable; OTSR, *Orthopaedics & Traumatology: Surgery & Research*; PRISMA, Preferred Reporting Items for Systematic Reviews and Meta-analyses; PROSPERO, International Prospective Register of Systematic Reviews.

### Level of Evidence and MQOE

Of the SRs, 6 were graded as Level III evidence and 9 were graded as Level IV evidence, as shown in Table 2. Compliance with PRISMA standards was mandated by 5 journals (71.4%) and was observed in 12 SRs (80%). Registering the SR with the PROSPERO database was advised although not mandated in 2 journals (28.6%) and was observed in 1 study (6.6%). All SRs reported on study funding; none declared a source of funding. All SRs reported on potential conflicts of interest, with 10 (66.7%) reporting no potential conflicts. The MQOE was assessed using the AMSTAR 2 rating; overall confidence in the results of the reviews was graded as critically low in 3 SRs (20%), low in 5 (33.3%), and moderate in 7 (46.7%).

## Discussion

The most important finding in this study was that nearly all SRs of the remplissage procedure are affected by spin. The most common forms of spin, which were found in over two-thirds of the included articles, were types 9 and 5, in which the conclusions claim the experimental treatment is of definite benefit “despite reporting bias” and “despite [a] high risk of bias in primary studies,” respectively. Thus, most of the evidence that has informed the change in clinical practice regarding uptake of the remplissage procedure is at risk of bias from spin, and this is known to have a significant impact on how study findings are interpreted by clinicians.^14^^,^^24^ Authors should design SRs with the AMSTAR 2 guidelines in mind, and journals should consider promoting these in their guidance for authors. Additionally, readers should always be cognizant of the potential for spin while reading articles and should critically appraise the full text to assess the relevant nuances.

The concept of spin was introduced to the medical literature to evaluate for reporting that may distort the readers’ interpretation.^13^ In recent years, this concept has been applied to the orthopaedic literature, with varying degrees of spin reported in SRs ranging from almost none to ubiquitous.^42^, ^43^, ^44^, ^45^, ^46^, ^47^ Regarding the evidence in shoulder surgery, spin has been reported to be present in 100% of SRs on superior capsular reconstruction,^48^ over 90% of SRs on balloon spacer placement,^43^ half of SRs on tendon transfer,^49^ and a third of SRs on rotator cuff tear and proximal humeral fracture treatment.^42^^,^^50^ The mean frequency of spin reported in these studies ranged from 1 to 3 instances, in keeping with the findings of our study. It is clear from the literature that SRs in this field are commonly affected by spin, particularly those of novel topics, and our study showed similar high rates of spin in SRs of the remplissage procedure.

MacDonald et al.,^7^ in a randomized controlled trial of ABR versus ABR with remplissage in patients with less than 15% glenoid bone loss, found a significantly lower rate of recurrence in the remplissage group (18% vs 4%, *P* = .027). They also reported a significantly reduced range of external rotation in abduction in the remplissage group at 12 months (85.7° vs 75.3°, *P* = .003) and non–statistically significant but potentially relevant greater level of self-reported limitation of sporting performance because of shoulder issues in the remplissage group (14.3% vs 20.7%). However, there is still no consensus on what constitutes critical glenoid bone loss in patients undergoing remplissage or at what point a bone block procedure is indicated.^9^^,^^51^^,^^52^ An ongoing randomized controlled trial comparing ABR with remplissage versus the Latarjet procedure in patients with 10% to 20% glenoid bone loss will provide further evidence.^53^ In the context of such uncertainty due to a lack of high-quality evidence, the spin observed in this SR has the potential to affect interpretations of the literature. An example of an overstated conclusion was that remplissage resulted in lower rates of recurrence compared with ABR or the Latarjet procedure.^38^ However, the meta-analysis compared ABR plus remplissage with the pooled results of ABR or the Latarjet procedure; given the vastly different recurrence rates observed between ABR and the Latarjet procedure, such a conclusion may not be appropriate.^54^ Likewise, a conclusion that remplissage significantly improved return-to-sport (RTS) rates minimized the finding of a 13% lower RTS rate in overhead or throwing athletes compared with the overall rate, with no comparison of RTS rates between remplissage and ABR for overhead athletes provided.^38^

The most commonly identified form of spin in this study was type 9—“conclusion claims the beneficial effect of the experimental treatment despite reporting bias”—which was reported in almost three-fourths of included SRs. The second most frequently noted form of spin was type 5—“conclusion claims the beneficial effect of the experimental treatment despite high risk of bias in primary studies”—which was found in two-thirds of included studies. A number of the included SRs did not report a risk-of-bias assessment, which is critical to give context to the findings.^32^^,^^39^^,^^41^ More than half of the included SRs had low or critically low AMSTAR 2 ratings; despite this, the mean 5-year impact factor of the journals in which these 8 reviews were published is currently 4.5 (range, 2.7-6.1), thus representing leading orthopaedic journals.^55^ The AMSTAR 2 assessment identified many factors that were broadly absent and, indeed, are rarely observed in modern orthopaedic publishing, including registering the trial protocol a priori, performing exhaustive searches for gray literature beyond the traditional databases, providing a complete list of the excluded studies, reporting the funding source of every included study, and performing an assessment of publication bias.^56^, ^57^, ^58^ However, it should be noted that there was a general trend toward higher MQOE ratings in more recent publications. No relation between potential conflicts of interest and spin was observed; no SR reported conflicting industry funding, nor was journal impact factor a predictor of the presence of spin. This is encouraging because it suggests that spin was not present owing to external influences and it reaffirms that a study should be assessed on its own merits given that journal impact factor alone is not a guarantee of high quality.^59^

### Limitations

This study is not without limitations. Despite grading by 2 independent reviewers who were trained in assessing spin prior to study commencement, there is still a subjective element to the grading of spin and this may be considered a limitation. Readers should be cognizant that there is no threshold for an acceptable level of spin or bias, nor does the presence of spin in a study completely invalidate all of its findings. The relevance of spin should be assessed on a case-by-case basis. Our study was intended to assess the remplissage procedure specifically, not to assess all literature on anterior shoulder instability, and some evidence regarding remplissage has not been assessed.

## Conclusions

Most SRs of the remplissage procedure are affected by the presence of spin. Favorable reporting was observed in the absence of definite findings, as was minimization of drawbacks for certain populations.

## Disclosures

The authors declare the following financial interests/personal relationships which may be considered as potential competing interests: C.S.K. reports a consulting or advisory relationship with Acumed, Restore3d, and Smith & Nephew and owns equity or stocks in GE Healthcare, Johnson & Johnson, Merck & Co, and Pfizer. J.F.D. reports board membership with American Academy of Orthopaedic Surgeons, *The American Journal of Sports Medicine*, American Orthopaedic Society for Sports Medicine, Arthroscopy Association of North America, and Society of Military Orthopaedic Surgeons. H.M. receives speaking and lecture fees from ConMed and DJO Surgical and reports board membership with Irish Orthopaedic Association. All other authors (T.R.D., M.S.D., T.K.M., M.W., E.T.H.) declare that they have no known competing financial interests or personal relationships that could have appeared to influence the work reported in this paper.
